# Interannual variation in the metazoan parasite communities of bigeye trevally *Caranx sexfasciatus* (Pisces, Carangidae)

**DOI:** 10.1051/parasite/2020001

**Published:** 2020-01-31

**Authors:** Juan Violante-González, Scott Monks, Yesenia Gallegos-Navarro, Nataly G. Santos-Bustos, Princessa J. Villalba-Vasquez, Jesús G. Padilla-Serrato, Griselda Pulido-Flores

**Affiliations:** 1 Facultad de Ecología Marina, Universidad Autónoma de Guerrero 39390 Acapulco Mexico; 2 Centro de Ciencias de Desarrollo Regional, Universidad Autónoma de Guerrero 39630 Acapulco Mexico; 3 Centro de Investigaciones Biológicas, Universidad Autónoma del Estado de Hidalgo 42000 Pachuca Mexico; 4 Conacyt – Facultad de Ecología Marina, Universidad Autónoma de Guerrero 39390 Acapulco Mexico

**Keywords:** Parasites, Marine fish, *Caranx sexfasciatus*, Acapulco, México

## Abstract

Parasite communities in *Caranx sexfasciatus* were characterized and analyzed to determine any interannual variations in structure and/or species composition. In total, 422 *C. sexfasciatus* were collected from Acapulco Bay, Mexico, between May 2016 and March 2019. Thirty-two taxa of metazoan parasites were identified: five Monogenea, thirteen Digenea, one Acanthocephala, one Cestoda, three Nematoda, seven Copepoda, and two Isopoda. Monogeneans were the most frequent and abundant parasite species in all sampling years. Parasite species richness at the component community level varied significantly from 8 (May 2016) to 25 (March 2019) and was similar to previous reports for other species of Carangidae. The component communities and infracommunities in *C. sexfasciatus* were characterized by low parasite species numbers, low diversity, and dominance of a single species (the monogenean *Neomicrocotyle pacifica*). Parasite community structure and species composition varied between sampling years and climatic seasons. Seasonal or local fluctuations in some biotic and abiotic environmental factors probably explain these variations.

## Abstract (fr)

Les communautés de parasites de *Caranx sexfasciatus* ont été caractérisées et analysées afin de déterminer si elles connaissent des variations interannuelles dans la structure et/ou la composition des espèces. Au total, 422 *C. sexfasciatus* ont été collectés dans la baie d’Acapulco, au Mexique, entre mai 2016 et mars 2019. Trente-deux taxons de parasites métazoaires ont été identifiés : cinq Monogenea, treize Digenea, un Acanthocephala, un Cestoda, trois Nematoda, sept Copepoda et deux Isopoda. Les monogènes étaient les espèces de parasites les plus fréquentes et les plus abondantes au cours de toutes les années d’échantillonnage. La richesse en espèces de parasites au niveau de la communauté des composants variait considérablement de 8 (mai 2016) à 25 (mars 2019) et était similaire aux rapports précédents pour d’autres espèces de Carangidae. Les communautés et infracommunautés de *C. sexfasciatus* étaient caractérisées par un faible nombre d’espèces de parasites, une faible diversité et la dominance d’une seule espèce (le monogène *Neomicrocotyle pacifica*). La structure des communautés de parasites et la composition des espèces variaient entre les années d’échantillonnage et les saisons climatiques. Les fluctuations saisonnières ou locales de certains facteurs environnementaux biotiques et abiotiques expliquent probablement ces variations.

## Introduction

Parasite communities in marine fish consist of ectoparasites (monogeneans, copepods, and isopods) and endoparasites (digeneans, cestodes, nematodes, and acanthocephalans). Both types of parasite exhibit different transmission strategies to infect their hosts; for example, ectoparasites are often transmitted directly between individual hosts through contact, whereas endoparasites often use trophic transmission routes [9, 26, 37]. Transmission strategies of parasites are linked to host behavior. For instance, ectoparasite populations may be more abundant on species of fish that form large schools than on solitary species [23, 29, 31], because the probability of a transmission stage (e.g., eggs, larvae) coming into contact with a host increases as host density rises.

Studies of parasite communities are conducted mainly at two different hierarchical levels: component community (between locations or host populations) and infracommunity (between individual hosts) [14, 17, 51]. In recent years, a central theme in parasite ecology has been the identification of the factors (biotic or abiotic) determining species richness and composition in these communities. However, an understanding is also needed of how consistent parasite community richness and species composition are in space and time. For example, spatial or temporal variation in community structure may indicate how important local environmental factors (abiotic and biotic conditions) are in structuring a parasite community, particularly when disparities are found between different host species [1, 24, 46, 50, 53]. There is still no consensus on whether marine parasite communities can exhibit temporal variations in structure and species composition. Some studies suggest that the infection levels of most parasite species do not experience significant changes over time, and that communities tend to be generally stable in species composition for long periods of time [4, 12, 16, 43]. Others indicate that even though parasite species composition may be relatively stable over time, a community can undergo substantial changes in structure due to variations in local environmental factors [14, 39, 51].

The bigeye trevally, *Caranx sexfasciatus* Quoy & Gaimard 1825, is an economically important pelagic fish species distributed widely along the eastern coast of the Pacific Ocean from CA, USA to Ecuador and the Galapagos Islands [2]. Bigeye trevally often form large stationary daytime schools, but usually tend to be solitary at night when feeding. Their diet consists mainly of fish, but the species also preys on squids and crustaceans [2]. Despite its ecological and economic importance, no information is available to date on its parasite fauna along the Pacific coast of Mexico. The present study objectives were (1) to characterize the metazoan parasite communities of *C. sexfasciatus*; and (2) to evaluate possible interannual variability in its parasite communities.

## Materials and methods

A total of 422 individuals of *C. sexfasciatus* were obtained from commercial fishermen over a four-year period (May 2016–March 2019) from Acapulco Bay (16°51′ N; 99°52′ W) in Guerrero, Mexico. The tropical Pacific region experiences two distinct climatic seasons: a rainy period from June to November (total precipitation ≈ 950 mm), and a dry period from December to May (total precipitation < 70 mm). In the years 2017 and 2019, a single sampling was carried out, but in a different climatic season: August (rainy season) and March (dry season), respectively. In 2016 and 2017, two samplings were carried out per year, which included both climatic seasons: May and October in 2016, and May and November in 2018. Records of surface temperatures of the ocean waters and salinity per sampling date were obtained from other studies carried out at the same location but not yet published. Multivariate El Niño index values (MEI) for each sampling date were obtained from the National Oceanic and Atmospheric Administration (NOAA: https://www.esrl.noaa.gov/psd/enso/mei/table.html). Fish were measured and weighed at the time of collection. A complete necropsy was made for each specimen and all parasites were collected from the internal and external organs [52]. Contents of the digestive tract were examined to identify prey items consumed by this species. Dietary items were identified to the family level when possible. Prey item analysis was carried out using the frequency of occurrence method [19, 30]. Parasites were identified to the lowest possible taxonomic level and vouchers of the most abundant and best-preserved specimens deposited in the Coleccion Nacional de Helmintos (CNHE), Instituto de Biologia, Universidad Nacional Autonoma de Mexico, Mexico City.

Infection levels for each parasite species were described using prevalence (percent of fish infected with a particular parasite species); mean abundance (mean number of individual parasites of a particular species per examined fish), expressed as the mean ± standard deviation (*SD*); and intensity (number of a particular parasite species per infected fish), expressed as range (minimum–maximum) [5, 6, 8]. Possible differences in infection levels between sampling years and climatic seasons were identified using *G*-tests [44], and a general linear model (GLM) for abundance. The dispersion index (DI = variance to mean abundance ratio) was applied to describe parasite dispersion patterns. The infracommunity index (ICI) [55], which describes the frequency of double and multiple infections by a single species of parasite in a distinct host (affinity level of a species of parasite; i.e. species with great tendency to join the infracommunity), was also calculated. Spearman’s correlation coefficient (*r*_*s*_) was used to determine possible relationships between total host length and abundance or DI values of each parasite species.

Analyses were done at the levels of component community (i.e. total parasite species in all fish collected at a sampling year) and infracommunity (i.e. total parasite species in each individual fish). Component community parameters included total species richness, total number of individuals of each parasite species, the Shannon–Wiener Index (*H*) as a measure of diversity, species evenness (equitability), and the Berger–Parker Index (BPI) as a measure of numerical dominance [25]. The qualitative Sorensen index and quantitative percentage of similarity (PS) index were used to evaluate similarity and difference in parasite community species composition between sampling dates. Differences between component community parameters were identified with Student *t* and *χ* ^2^ tests.

Infracommunities were described in terms of mean number of parasite species per host, mean number of individuals of each species, and the mean Brillouin Diversity Index (*H*′) value per host. The multivariate general linear model (GLM) was used to identify possible differences in infracommunity parameters (dependent variables) between sampling years and climatic seasons (predictor variables); fish body size (total length) was used as a covariate to control for the influence of host body size. The significance of all statistical analyses was established at *p* < 0.05, unless stated otherwise. A multivariate analysis (principal component analysis, PCA) was applied to identify factors that influenced parasite infracommunity species richness and diversity. The predictor variables used were: Fulton’s condition factor (*K*_*n*_) [13]; host diet diversity (calculated as the diversity of items consumed by host populations at each sampled date, through the use of the Shannon–Wiener index at the family level); number of endoparasite and ectoparasite species; surface temperature; salinity; MEI values; sampling year; and climatic season. The Kaiser–Meyer–Olkin (KMO) test of sampling adequacy for each variable in the model, as well as the Bartlett sphericity test, which evaluates the possibility that there is redundancy among variables were applied. The variance maximizing rotation method was applied to produce the two ordination axes. Discriminant function analyses based on Mahalanobis distances were used to identify possible differences in parasite community structure between sampling years. The probability of correct classification expected by chance alone of fishes to any of the four sampling years was calculated using the proportional chance criterion, which is a simple method to account for differences in sample sizes between the groups being compared [36]. Only parasite species with a prevalence >10% in at least one of the sampling years (a component species; *sensu* [7]) were included in this analysis.

## Results

### Species composition

**Figure 1:**
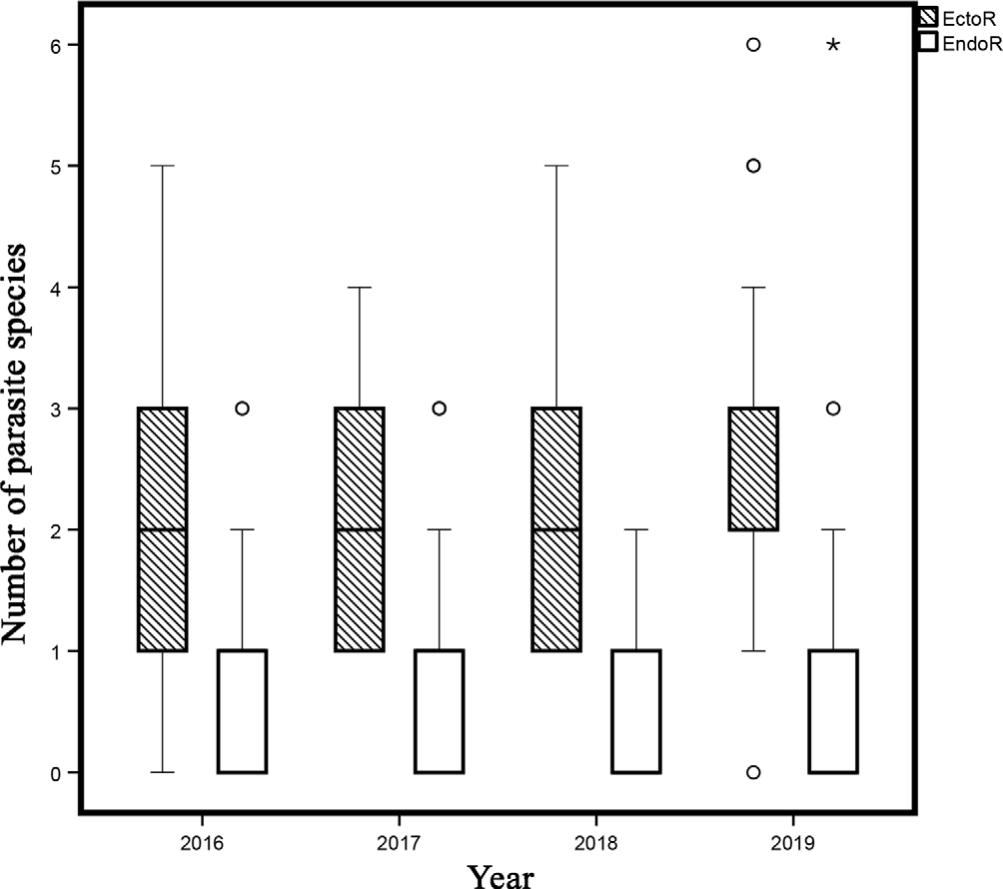
Box plot of species number of ectoparasites (monogenean and crustacean), and endoparasites (larvae and adults) in *Caranx sexfasciatus* from Acapulco Bay. The bottom and top of the boxes represent the lower and upper quartiles respectively; the median is shown as a line through the centre of the boxes; outliers are indicated with circles for ectoparasites and asterisks for endoparasites.

Thirty-two taxa of metazoan parasites (23 of helminths and nine of Crustacea) were recovered and identified (9866 individual parasites) from 422 individuals of *C. sexfasciatus* collected from Acapulco Bay, Mexico. Five species of Monogenea (adults), 13 Digenea (12 adults and one metacercaria), one Acanthocephala (adult), three Nematoda (one adult and two larvae), seven Copepoda, and two species of Isopoda were collected (Table 1). Species richness was highest among the digeneans, representing 37.1% of the total species, followed by copepods (20%). Based on infection site, 14 species of parasite were classified as ectoparasites and 18 as endoparasites. The numbers of species of ectoparasite varied significantly from 2.02 ± 0.90 in 2016 to 2.41 ± 1.20 in 2019 (ANOVA *F*_3,405_ = 3.23, *p* < 0.05) (Fig. 1). Eleven species, *Neomicrocotyle pacifica* Yamaguti 1968, *Protomicrocotyle manteri* Bravo-Hollis 1966, *Pseudomazocraes selene* Hargis 1957, *Bucephalus varicus* Manter 1940, *Ectenurus virgulus* Looss 1910*, Synaptobothrium apharei* Yamaguti 1970, Tetraphyllidea gen. sp., *Anisakis* sp., *Caligus alalongae* Krøyer 1863, *Ca. robustus* Bassett-Smith 1898, and *Lernanthropus ilishae* Chin 1948 occurred in the parasite communities of *C. sexfasciatus* in all sampling years (Table 1).

**Table 1:** Parasite infection parameters for *Caranx sexfasciatus* from Acapulco Bay, Mexico.

Parasite taxa	Site on host	Accession number^a^	Year	*n* b	Prevalence (%)	Total^c^	Abundance^d^	Range of intensity^e^	DI^f^	ICI^g^
Digenea										
*Bucephalus margaritae* Ozaki & Ishibashi, 1934	Intestine	10,997	2017	97	2.06	3	0.03 ± 0.71	1–2	0.33	0.007
			2018_M_	63	4.76	3	0.05	0–1		0.021
			2019	110	3.64	10	0.09 ± 2.4	1–6	2.27	0.015
*Bucephalus varicus* h Manter, 1940	Intestine	10,998	2016_M_	65	40*	81	1.25 ± 2.2	1–9	1.52	0.146
			2016_O_	36	27.78	67	1.86 ± 7.4*	1–25	8.13	0.109
			2017	97	24.74	70	0.72 ± 3	1–12	3.04	0.086
			2018_M_	63	14.29	26	0.41 ± 2.8	1–9	2.72	0.064
			2018_N_	51	3.92	2	0.04	0–1		0.016
			2019	110	19.09	157	1.43 ± 8.6	1–31	9.89	0.081
*Neoapocreadium* cf. *caranxi*	Intestine		2017	97	1.03	1	0.01	0–1		0.004
Didymozoid larvae	Intestine		2017	97	4.12	6	0.06 ± 0.6	1–2	0.22	0.014
			2018_N_	51	1.96	2	0.04	0–2		0.008
			2019	110	2.73	3	0.03	0–1		0.012
*Ectenurus virgulus* h Looss, 1910	Intestine	11,000	2016_M_	65	29.23*	38	0.58 ± 3	1–14	4.39	0.107
		11,001	2016_O_	36	25.00	35	0.97 ± 3.8	1–13	3.69	0.098
			2017	97	20.62	32	0.33 ± 1.1	1–5	0.75	0.071
			2018_N_	51	9.80	10	0.20 ± 1.7	1–5	1.50	0.041
			2019	110	13.64	38	0.35 ± 3.2	1–14	4.11	0.058
*Hirudinella ventricosa* (Pallas, 1774) Baird, 1853	Intestine		2017	97	5.15	5	0.05	0–1		0.018
			2018_N_	51	1.96	1	0.02	0–1		0.008
			2019	110	0.91	1	0.01	0–1		0.004
*Lecithocladium excisum* (Rudolphi, 1819) Lühe, 1901	Intestine		2019	110	1.82	3	0.03 ± 0.7	1–2	0.33	0.008
*Manteria brachyderus* (Manter, 1940) Caballero & Caballero, 1950	Intestine		2018_N_	51	1.96	1	0.02	0–1		0.008
*Mecoderus oligoplitis* Manter, 1940	Intestine		2019	110	0.91	1	0.01	0–1		0.004
*Pseudopecoeloides carangi* (Yamaguti, 1938) Yamaguti, 1940	Intestine	11,002	2018_M_	63	1.59	1	0.02	0–1		0.007
*Stephanostomum ditrematis* (Yamaguti, 1939) Manter, 1947	Intestine		2018_N_	51	1.96	1	0.02	0–1		0.008
			2019	110	1.82	2	0.02	0–1		0.008
*Stephanostomum carangis* (Yamaguti, 1951) Caballero, 1952	Intestine		2017	97	1.03	1	0.01	0–1		0.004
*Synaptobothrium apharei* (Yamaguti, 1970) Leon-Regagnon, Perez-Ponce de Leon & Lamothe-Argumedo, 1997	Intestine	11,004	2016_O_	36	2.78	2	0.06	0–2		0.011
		11,005	2017	97	4.12	8	0.08 ± 1.4	1–4	1	0.014
			2018_N_	51	7.84	6	0.12 ± 1	1–3	0.67	0.033
			2019	110	5.45	7	0.06 ± 0.4	1–2	0.14	0.023
Monogenea										
*Heteromicrocotyla carangis* Yamaguti, 1953	Gills		2019	110	0.91	1	0.01	0–1		0.004
*Neobenedenia melleni* (MacCallum, 1927) Yamaguti, 1963	Gills		2019	110	0.91	1	0.01	0–1		0.004
*Neomicrocotyle pacifica* h (Meserve, 1938) Yamaguti, 1968	Gills	10,992	2016_M_	65	86.15	899	13.83 ± 15.5	1–57	15.06	0.314
		10,993	2016_O_	36	44.44	262	7.28 ± 21.2	2–76	27.19	0.174
			2017	97	91.75*	2271	23.41 ± 19.5	1–103	14.88	0.317
			2018_M_	63	85.71	589	9.35 ± 10.9	1–61	10.85	0.386
			2018_N_	51	62.75	718	14.08 ± 39.1	1–184	67.98	0.260
			2019	110	80.91	2818	25.62 ± 31.4*	1–142	31.10	0.343
*Protomicrocotyle manteri* h Bravo-Hollis, 1966	Gills	10,994	2016_M_	65	63.08	199	3.06 ± 5.1	1–20	5.26	0.230
		10,995	2016_O_	36	47.22	229	6.36 ± 18.3	1–76	24.98	0.184
			2017	97	45.36	114	1.18 ± 2.6	1–13	2.66	0.157
			2018_M_	63	46.03	67	1.06 ± 1.5	1–6	1.02	0.207
			2018_N_	51	56.86	173	3.39 ± 8.4	1–45	11.82	0.236
			2019	110	60.00	265	2.41 ± 4.3	1–24	4.62	0.254
*Pseudomazocraes selene* h Hargis, 1957	Gills	10,996	2016_M_	65	1.54	1	0.02	0–1		0.006
			2016_O_	36	13.89	13	0.36 ± 3.1*	1–8	3.58	0.054
			2017	97	1.03	1	0.01	0–1		0.004
			2018_M_	63	3.17	9	0.14 ± 3.5	1–7	2.78	0.014
			2018_N_	51	19.61*	16	0.31 ± 1	1–4	0.58	0.081
			2019	110	12.73	19	0.17 ± 0.5	1–2	0.18	0.054
Cestoda										
Tetraphyllidea gen. sp.^h^	Intestine		2016_O_	36	16.67*	18	0.50 ± 2.7	1–8	2.40	0.065
			2017	97	3.09	3	0.03	0–1		0.011
			2018_N_	51	7.84	8	0.16 ± 0.8	1–3	0.33	0.033
			2019	110	0.91	1	0.01	0–1		0.004
Acanthocephala										
*Rhadinorhynchus* sp.	Intestine		2018_N_	51	1.96	7	0.14	0–7		0.008
			2019	110	1.82	2	0.02	0–1		0.008
Nematoda										
*Anisakis* sp.^h^ larvae	Mesentery		2016_M_	65	4.62	6	0.09 ± 1.7	1–4	1.50	0.017
			2016_O_	36	5.56	3	0.08 ± 0.7	1–2	0.33	0.022
			2017	97	16.49*	31	0.32 ± 1.4	1–6	1.06	0.057
			2018_M_	63	6.35	4	0.06	0–1		0.029
			2019	110	6.36	10	0.09 ± 0.8	1–3	0.43	0.027
*Contracaecum* sp. larvae	Mesentery		2018_N_	51	1.96	1	0.02	0–1		0.008
*Procamallanus* sp.	Intestine		2019	110	0.90	1	0.01	0–1		0.004
Copepoda										
*Bomolochus* sp.	Gills	123	2016_O_	36	2.78	1	0.03	0–1		0.011
*Caligus alalongae* h Krøyer, 1863	Gills	120	2016_M_	65	9.23	6	0.09	0–1		0.034
			2016_O_	36	8.33	3	0.08	0–1		0.033
			2017	97	22.68*	54	0.56 ± 1.8*	1–7	1.39	0.078
			2018_M_	63	3.17	2	0.03	0–1		0.014
			2018_N_	51	1.96	1	0.02	0–1		0.008
			2019	110	0.91	1	0.01	0–1		0.004
*Caligus chorinemi* Krøyer, 1863	Gills	121	2017	97	2.06	2	0.02	0–1		0.007
			2019	110	8.18	10	0.09 ± 0.3	1–2	0.10	0.035
*Caligus robustus* h Bassett-Smith, 1898	Gills	122	2016_M_	65	40.00	41	0.63 ± 0.7	1–4	0.31	0.146
			2016_O_	36	44.44	30	0.83 ± 1.3	1–5	0.84	0.174
			2017	97	32.99	49	0.51 ± 0.6	1–3	0.21	0.114
			2018_M_	63	38.10	51	0.81 ± 1.8	1–7	1.49	0.172
			2018_N_	51	49.02	44	0.86 ± 1.2	1–5	0.77	0.203
			2019	110	50.91	89	0.81 ± 0.8	1–4	0.36	0.216
*Caligus mutabilis* Wilson, 1905	Gills		2017	97	1.03	1	0.01	0–1		0.004
*Ergasilus* sp.	Gills		2016_O_	36	8.33	4	0.11 ± 0.6	1–2	0.25	0.033
			2018_N_	51	5.88	3	0.06	0–1		0.024
			2019	110	1.82	2	0.02	0–1		0.008
*Lernanthropus ilishae* h Chin, 1948	Gills	124	2016_O_	36	5.56	2	0.06	0–1		0.022
			2017	97	9.28	11	0.11 ± 0.7	1–3	0.36	0.032
			2018_M_	63	17.46*	13	0.21 ± 0.4	1–2	0.14	0.079
			2018_N_	51	1.96	1	0.02	0–1		0.008
			2019	110	15.45	19	0.17 ± 0.3	1–2	0.10	0.065
Isopoda										
*Gnathia* sp.	Gills		2019	110	2.73	3	0.03	0–1		0.012
			2018_M_	63	1.59	36	0.57	0–36		0.007
*Rocinella signata* Schioedte & Meinert, 1879	Gills	125	2016_O_	36	2.78	1	0.03	0–1		0.011
			2018_N_	51	1.96	1	0.02	0–1		0.008
			2019	110	0.91	1	0.01	0–1		0.004

a Specimens deposited at the CNHE (Colección Nacional de Helmintos, Instituto de Biología, Universidad Nacional Autónoma de México).

b Number of fish examined.

c Total number of individual parasites collected.

d Number of parasites per fish (mean ± SD).

e Minimum to maximum number of parasites present.

f Variance to mean ratio.

g Infracommunity index.

h Component species.

Subscripts to the right of the sampling year indicate the sampling month: M = May, O = October, N = November.

*Significantly different measurements of prevalence (*G*-test) and abundance (two-way ANOVA) (*p* < 0.05).

### Interannual variation in infection levels

For eight of these perennial species (the exceptions were *Pr. manteri*, *S. apharei* and *Ca. robustus*), prevalence varied significantly between sampling years, though no clear pattern was observed in variation. For the parasites *B. varicus* (*G* = 35.1, *p* < 0.05) and *E. virgulus* (*G* = 12.9, *p* < 0.05) infection percentages were highest in May 2016. For the Tetraphyllidea cestode, prevalence was highest during October 2016. The species *N. pacifica* (*G* = 21.8, *p* < 0.05), *Anisakis* sp. (*G* = 12.1, *p* < 0.05) and *Ca. alalongae* (*G* = 42.3, *p* < 0.05) exhibited their highest percentages of infection in 2017. For *L. ilishae* (*G* = 17.1, *p* < 0.05), the highest percentage occurred in May 2018, while for *Ps. selene* (*G* = 34.9, *p* < 0.05), it was in November 2018 (Table 1).

In contrast to the percentage infection results, only five species (*N. pacifica* [GLM; *F*_3,406_ = 7.92, *p* < 0.05], *Ps. selene* [GLM; *F*_3,406_ = 3.05, *p* < 0.05], *B. varicus* [GLM; *F*_3,406_ = 3.10, *p* < 0.05], Tetraphyllidea cestode [GLM; *F*_3,406_ = 5.49, *p* < 0.05], and *Ca. alalongae* [GLM; *F*_3,406_ = 5.31, *p* < 0.05]) exhibited significant interannual variation in abundance. Abundance for *B. varicus*, *Ps. selene* and Tetraphyllidea larvae was highest in October 2016. For *Ca. alalongae*, it was highest in 2017, while for *N. pacifica* it was highest in 2019 (Table 1). Prevalence values were generally positively correlated with mean abundance values, indicating that the most prevalent species also were the most abundant (*r*_*s*_ = 0.946, *p* < 0.01).

### Spatial dispersion

The dispersion index (DI) values indicated that at least 10 species (31%) exhibited an aggregated dispersion pattern in one or more sampling years (Table 1). Higher mean aggregation values (DI > 8) occurred in several years for the monogeneans *N. pacifica* and *Pr. manteri* (Table 1). The DI values of each parasite species correlated positively with its prevalence (*r*_*s*_ = 0.824, *p* < 0.01), total number of individuals (*r*_*s*_ = 0.878, *p* < 0.01), and mean abundance (*r*_*s*_ = 0.871, *p* < 0.01) values, but not with host body size or sample size (*p* > 0.05).

### Host diet composition

The alimentary spectrum of the fish included twelve prey items, of which smaller fish (36%), crab larvae (24.5%) and penaeid larvae (15.6%) represented the largest proportions. Additonal prey items accounted for 24% of the diet, and included amphipods, polychaetes, mollusk larvae, isopods, stomatopods, pistol crabs, and copepods. Diet composition varied between sampling years (*t* = 3.10, *p* < 0.05). For example, in May 2016 diet composition was less varied (*H* = 0.68, Table 2), with crab larvae being the main prey item. Feeding habits also changed with fish body size; larger individuals consumed higher percentages of fish while smaller ones fed on higher percentages of crustaceans and other prey.

**Table 2:** Characteristics of the parasite component communities and infracommunities of *Caranx sexfasciatus* from Acapulco Bay, Mexico.

Year	Date	SST °C	Salinity (ppt)	No. fish	Length (cm)	Diet	Component communities						Infracommunities		
							Species richness	No. of parasites	BPI	Dominant species	% of dom. infra.	*H*	Mean number of species	Mean number of parasites	Mean value of Brillouin Index
2016a	May ^d^	28	34.9	65	23.9 ± 4.5	0.68	8	1271	0.707	Neom	73.8	0.90	2.70 ± 1.10	19.5 ± 16.79	0.70 ± 0.41
2016b	Oct ^r^	30	34.4	36	19.0 ± 1.3	0.90	14	670	0.391	Neom	13.9	1.15	2.56 ± 1.83	18.61 ± 27.86	0.71 ± 0.58*
2017	Aug ^r^	30	34.6	97	28.8 ± 4.5	0.96	18	2663	0.853	Neom	88.7	1.25	2.89 ± 1.30	27.4 ± 20.1.37	0.56 ± 0.43
2018a	May ^d^	29	34.8	63	26.7 ± 3.1	0.70	11	801	0.735	Neom	74.6	1.04	2.22 ± 1.30	12.71 ± 11.50	0.53 ± 0.48
2018b	Nov ^r^	30	33.6	51	24.0 ± 3.5	1.0	18	996	0.721	Neom	47.0	1.25	2.41 ± 1.25	19.53 ± 33.98	0.64 ± 0.44
2019	Mar ^d^	28	34.1	110	26.5 ± 5.9	1.1	25	3465	0.813	Neom	64.5	1.40	2.96 ± 1.54*	31.50 ± 30.35*	0.61 ± 0.49

SST = sea surface temperature, Length = total length, Diet = diet variety, BPI = Berger-Parker Index; % of dom. infra = percentage of dominated infracommunities, H = Shannon-Wiener diversity index, Neom = *Neomicrocotyle pacifica*. *Significant at *p* < 0.05. Letters as superscripts represent codes of assignment ranges of locations and climatic seasons (d = dry, r = rainy) used in the multivariate statistical analysis.

### Component community

**Figure 2:**
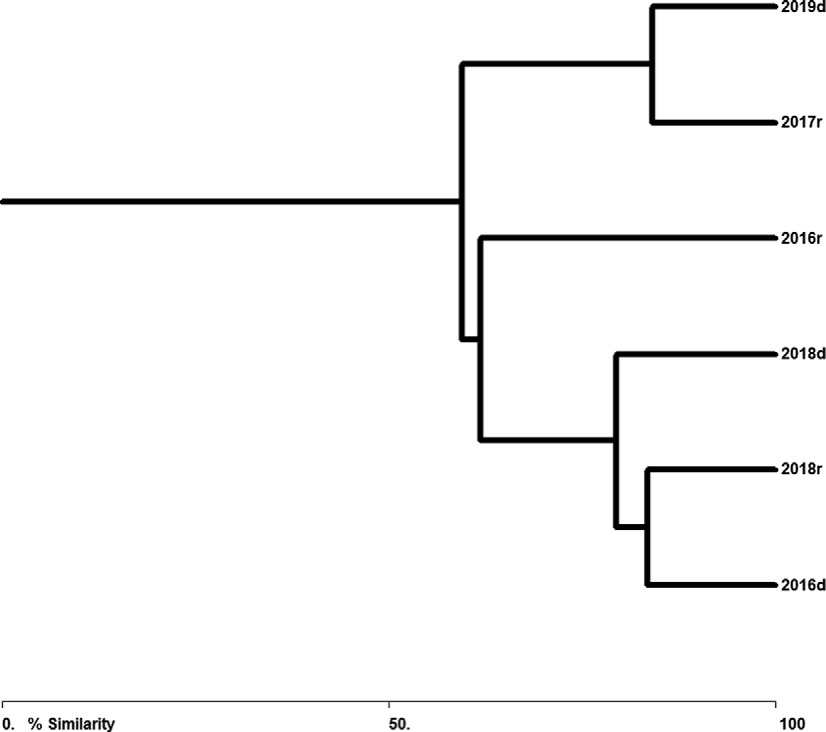
Similarity percentages (Bray-Curtis index) for parasite communities of *Caranx sexfasciatus* between sampling years. d = dry season, r = rainy season.

Species richness of parasite by sampling year (Table 2) varied widely from eight (May 2016) to 25 (March 2019) (*χ* ^2^ = 11.6, *p* < 0.05). No correlation was observed between sample size and species richness at this level, indicating that the different sample sizes used in the analyses had no effect on the results. The total number of individual parasites ranged from 670 (October 2016) to 3465 (March 2019) (*χ* ^*2*^ = 3,997.2, *p* < 0.05). The only dominant species was the monogenean *N. pacifica* (Table 2), although its degree of dominance varied significantly between sampling years (*t* = 4.70, *p* < 0.01). Shannon–Wiener diversity index values were generally low, ranging from 0.90 (October 2016) to 1.40 (March 2019), but varied between sampling years (*t* = 3.68, *p* < 0.01). Similarity between component communities was slightly low overall (<65%, Fig. 2) and was higher at the qualitative level (mean = 64.05%) than at the quantitative level (mean = 56.15%).

### Infracommunity

The body size of individuals of *C. sexfasciatus* varied from 19.0 ± 1.3 cm (October 2016) to 28.8 ± 4.5 cm (August 2017) (Table 2), and it differed significantly between sampling years (ANOVA *F*_3,402_ = 32.3, *p* < 0.01). Mean species richness of parasites ranged from 2.22 ± 1.30 to 2.96 ± 1.54, and the mean number of individual parasites per fish from 12.71 ± 11.5 to 31.50 ± 30.35 (Table 2). The Brillouin diversity index (*H*′) values varied from 0.53 ± 0.48 to 0.71 ± 0.58. The mean number of species (GLM; *F*_3,406_ = 3.95, *p* < 0.01) and the mean number of individuals (GLM; *F*_3,406_ = 10.72, *p* < 0.01) were highest in March 2019 (Table 2), although mean diversity (*H*′) did not exhibit significant variation between sampling years (*p* > 0.05). Dominance of the monogenean *N. pacifica* at the infracommunity level was very similar to that at the component level (Table 2). Considering all samples, the mean number of species (*r*_*s*_ = −0.368, *p* < 0.01), and the mean diversity (*r*_*s*_ = −0.098, *p* < 0.05) exhibited negative correlations with host body size of fish, while the mean number of individual parasites was positively correlated (*r*_*s*_ = 0.313, *p* < 0.01). The infracommunity index values (ICI) indicated that *N. pacifica*, *Pr. manteri*, and *Ca. robustus* had the highest number of double or multiple co-occurrences with other parasite species (ICI mean > 0.15) in all the sampling years (Table 1).

### Multivariate analyses

**Figure 3:**
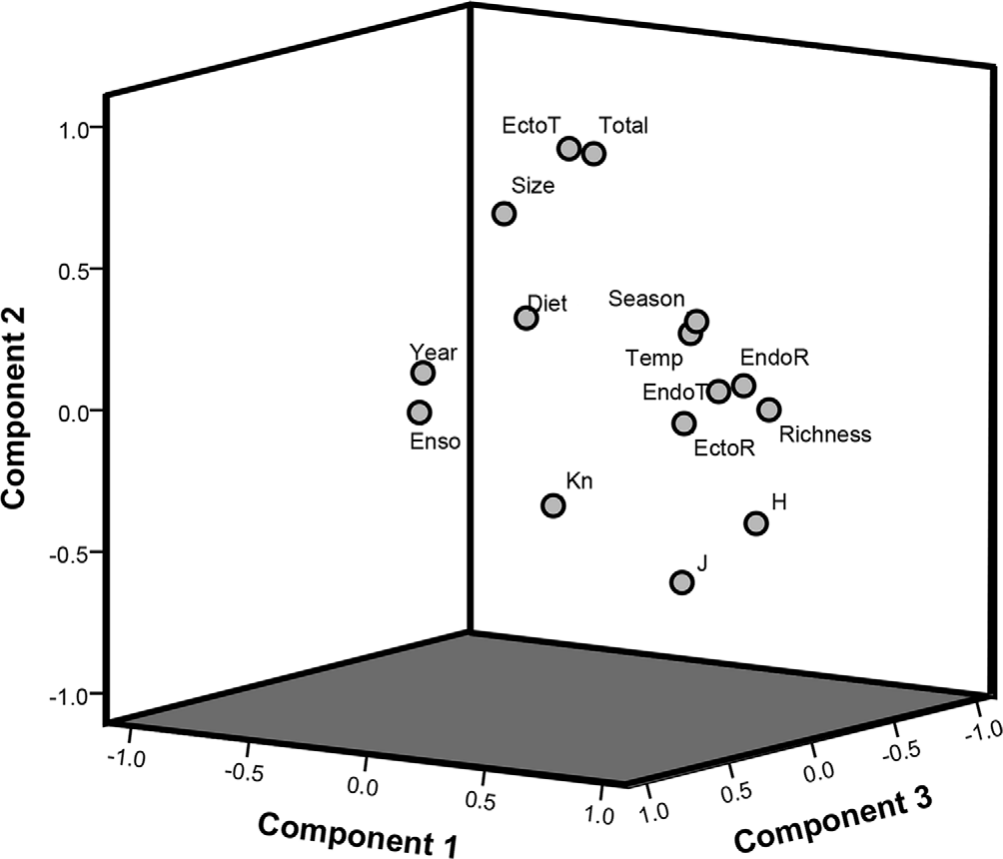
Scatter plot of the principal component analysis (PCA) of factors that influence the species richness and diversity of the parasite infracommunities of *Caranx sexfasciatus*, from Acapulco Bay. Predictor variables: Year = sampling year, Season = climatic season (dry/rainy), Temp = surface temperature values, Enso = Multivariate El Niño index, Size = host body size (total length), *K*_*n*_ = condition factor, Diet = host diet variety, EndoR = number of endoparasite species, EctoR = number of ectoparasite species, EndoT = total number of endoparasites per host, EctoT = total number of ectoparasites per host. Infracommunity parameters: Richness = number of parasite species per infracommunity, Diversity = Brillouin diversity index values, Load = total number of parasites per infracommunity, Evenness = species evenness.

The KMO (0.476) and the Bartlett’s tests results (*χ* ^2^ = 2,944.2; *df* = 45, *p <* 0.001) for the PCA, applied to identify the influence of biotic and abiotic factors on parasite infracommunity structure (Fig. 3), indicated that there is a sufficient relationship between the number of variables studied and the sample sizes, confirming the relevance of the PCA. The first three component variables generated by the model explained 61.9% of total variance, contributing 24.9% (eigenvalue = 3.74), 19.8% (eigenvalue = 2.97), and 17.2% (eigenvalue = 2.58). The first variable suggests that the richest and most diverse infracommunities among all the sampled years were characterized by a significant number of ectoparasite and endoparasite component species, with more homogeneous species abundances. The second variable indicated that both the total number of ectoparasites and the parasite load per infracommunity were highest in larger hosts with a more varied diet. The third variable associated host diet with the environmental factors that were studied (Table 3). This variable indicated that host diet was more varied during the dry season when water surface temperature was slightly cooler (as suggested by the negative correlations), which occurred mainly during the final sampling year (Tables 2 and 3).

**Table 3:** Summary of the principal component analysis (PCA) on biotic and abiotic factors that influence the diversity and species richness of the infracommunities of *Caranx sexfasciatus*. The three principal component axes are indicated by PC1, PC2 and PC3, respectively. Variables that contribute to the total explained variance in each PC are in bold. Eigenvalues, percentage of variance, and accumulative variance are shown at the end of the table. Communality = total influence of the variable with respect to all other associated variables. Uniqueness = percentage of variability that is not predicted by the variable in the model.

				Communality	Uniqueness
Variables	PC1	PC2	PC3	*R* ^2^	% not predicted
Richness	**0.961**	0.050	0.045	0.928	0.072
Diversity	**0.892**	−0.355	0.024	0.922	0.078
EctoR	**0.709**	0.005	0.200	0.543	0.457
EndoR	**0.705**	0.085	−0.167	0.532	0.468
EndoT	**0.667**	0.075	−0.070	0.455	0.545
Evenness	**0.594**	−0.590	0.048	0.703	0.297
EctoT	0.148	**0.904**	0.095	0.848	0.152
Load	0.244	**0.894**	0.082	0.865	0.135
Size	−0.171	**0.633**	0.033	0.431	0.569
*K*_*n*_	0.123	**−0.351**	0.156	0.162	0.838
Diet	0.135	**0.339**	**0.336**	0.246	0.754
Temp	0.007	0.102	**−0.842**	0.720	0.280
Enso	0.027	0.066	**0.892**	0.692	0.308
Season	0.073	0.159	**−0.785**	0.646	0.354
Year	−0.016	0.189	**0.746**	0.592	0.408
Eigenvalue	3.74	2.97	2.58		
% total variance	24.9	19.8	17.18		
Accumulative variance	24.9	44.7	61.9		

Predictor variables: Year = sampling year, Season = climatic season (dry/rainy), Temp = surface temperature values, Enso = Multivariate El Niño index, Size = host body size (total length), *K*_*n*_ = condition factor, Diet = host diet variety, EndoR = number of endoparasite species, EctoR = number of ectoparasite species, EndoT = total number of endoparasite per host, EctoT = total number of ectoparasite per host. Infracommunity parameters: Richness = number of parasite species per infracommunity, Diversity = Brillouin diversity index values, Load = total number of parasites per infracommunity, Evenness = species evenness.

**Figure 4:**
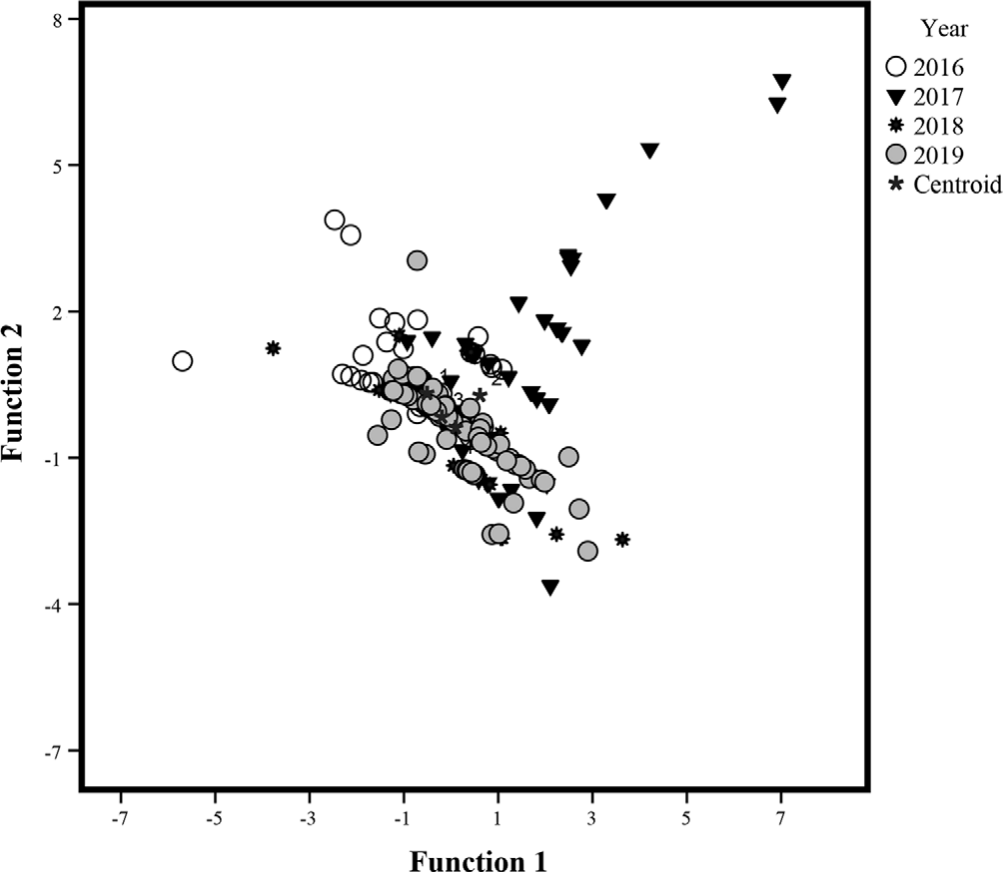
Graphic of multivariate discriminant analyses for parasite communities of *Caranx sexfasciatus*. The symbols represent each one of the fish examined in each sampling year. Centroid = mean group.

In the model constructed to identify possible interannual differences in parasite community structure, the first two discriminant variables explained 86.9% of the total variance, contributing 56.2% (eigenvalue = 0.164) and 30.6% (eigenvalue = 0.090). A significant overall group effect was observed (Wilks’ lambda = 0.759, *p* < 0.001). Individual fish were mainly distributed along the first axis (Fig. 4). Dimensionality tests showed that the four sampling years were significantly different in both dimensions (χ^2^ = 110.37, *df* = 15, *p* < 0.001). Each fish was correctly assigned to one of the four sampling years with a 41.1% accuracy, more than double that achieved by chance alone (16%).

Of the nine component species (i.e. prevalence > 10%), only five were accepted by the model (Table 4) based on their lower Wilks’ lambda values. High abundance of the monogenean *Pr. manteri* and lower abundance of the digenean *E. virgulus* were characteristic of hosts collected in 2016. The monogenean *N. pacifica* and the copepod *L. ilishae* effectively functioned to identify hosts that were collected in 2019, while the copepods *Ca. alalongae* and *L. ilishae* effectively distinguished between hosts collected in 2017 and 2018.

**Table 4:** Discriminant analysis classification showing the numbers and percentages of the *Caranx sexfasciatus* classified in each sampling year (rows correspond to group memberships). The bottom of the table shows the matrix of classification coefficient values for parasites that allowed differentiation among sampling years; coefficient values in bold indicate the importance of each species of parasite in distinguishing among years.

Sampling year	2016	2017	2018	2019	Percent
2016	22	7	57	10	22.9
2017	5	22	41	28	22.9
2018	3	3	81	19	76.4
2019	8	5	53	42	38.9
Parasite					
*Protomicrocotyle manteri*	**0.135**	0.030	0.063	0.068	
*Neomicrocotyle pacifica*	0.021	0.044	0.024	**0.048**	
*Ectenurus virgulus*	**0.349**	0.112	0.056	0.032	
*Caligus alalongae*	0.214	**1.239**	0.052	0.089	
*Lernanthropus ilishae*	−0.070	0.961	**0.982**	**1.389**	

## Discussion

### Parasite community species composition

All the present parasite species records (23 helminths and nine crustaceans) are new geographical records for *C. sexfasciatus* on the Pacific coast of Mexico (Table 1). Ectoparasites (five monogeneans, seven copepods, and two isopods) numerically dominated the parasite communities, representing 92.71% of the total number of individual parasites recovered. This predominance is probably due to the monogeneans’ transmission strategies (direct contact), increasing their transmission probabilities especially in gregarious fish such as *C. sexfasciatus* [27, 42]. Eight of the reported monogenean species (including *N. pacifica*, *Pr. manteri*, and *Ps. selene*) have been recorded in Carangidae from the Pacific coast of Mexico [32]. Copepods are a highly diverse group that mainly parasitize marine fish [22, 28, 45]; however, this crustacean group has received limited attention in Mexico [14]; in the present study, they were the second most diverse group and represented 22% of the total taxa recovered (Table 1).

### Interannual variation in parasite infection levels

At least five species of parasite (two endoparasites – *B. varicus* and a tetraphyllidean cestode, and three ectoparasites – *N. pacifica, Ps. selene* and *Ca. alalongae*) exhibited significant interannual differences in infection levels (Table 1). Endoparasites often are trophically transmitted, so variations in infection levels can be explained by interannual changes in host diet and availability of prey harboring infective stages [16, 18, 23, 38]. The population density and gregarious behavior (schooling behavior) of *C. sexfasciatus* [2] may be a better explanation for the annual variation observed in the three species of ectoparasites. Fish that form large schools facilitate ectoparasite transmission, particularly parasites with a direct transmission cycle such as monogeneans and copepods [14, 23, 39, 41, 51].

### Dispersion pattern

The DI values indicated that at least 31% of the species of parasites exhibited aggregate dispersion in one or more of the sampling years (Table 1). Aggregation is considered a typical dispersion pattern of parasites in marine fish [3, 18, 37]. However, the widely variable aggregation levels of five helminth species (the monogeneans *N. pacifica* and *Pr. manteri*; the digeneans *B. varicus* and *Ectenurus virgulus*; and the nematode *Anisakis* sp.) suggest the existence of differences in host exposure rates to parasites. Differing exposure rates could be due to the influence of local environmental factors [35], or the differences in host feeding behavior revealed in the diet analysis. In contrast, some contact-transmitted parasites such as monogeneans, exhibit high host specificity and can be more abundant in larger fish that form schools [23, 29], like *C. sexfasciatus*. Some studies also suggest that DI values can be influenced by sample size [33, 35], but there was no positive correlation between DI values and sample sizes in the present analysis.

### Component communities

The parasite communities in *C. sexfasciatus* exhibited similar patterns at the component and infracommunity levels: low species numbers (2.22–2.96 species on average per host), low species diversity, and dominance by a single species (the monogenean *Pr. manteri*, Table 2). Species richness at the component level (8–25 species, Table 2) is similar to that reported previously in other *Caranx* species in the Americas, such as *C. hippos* (19 species), *C. latus* (17 species), and *C. caballus* (18 species) [15, 21]. However, overall parasite fauna of *C. sexfasciatus* included 32 species across the four sampling years. The broad geographical distribution of *C. sexfasciatus* may explain this greater overall richness since hosts with a broad geographical distribution are exposed to large numbers of parasite species by interaction with myriad intermediate host species throughout their distribution range [40]. Species composition similarity between the parasite communities (Fig. 2) was considered generally low at both levels (qualitative and quantitative levels: <65%), compared to the similarity recorded for parasite communities of other marine fish examined on the Mexican Pacific coast, such as *Caranx caballus* 78.5% [14], *Oligoplites altus* 85% [39], or *Parapsettus panamensis* 76% [51]. This low similarity can be attributed to the fact that 47% (15 species: six ectoparasites and nine endoparasites) of the identified parasite species occurred only in one or two sampling years (Table 1). This suggests that annual variations in biotic (e.g. diet, body size, availability of larva-infected prey) and/or abiotic environmental factors [14, 23, 39, 49, 51], may have been responsible for the low similarity between the parasite communities of *C. sexfasciatus* observed in the present study.

### Infracommunities

Several biotic and abiotic factors are known to strongly influence species richness and diversity in the parasite communities of marine fish over time [14, 23, 34, 39, 48, 51]. The PCA results (Fig. 3) indicated that infracommunity species richness and diversity depended heavily on the occurrence of a distinctive set of component species (both ecto- and endoparasite species). However, these component species’ frequency or abundance varied between sampling years (Table 1). Temporal variations in parasite infracommunity structure and species composition have been attributed to variations in the prevalence and/or abundance of some dominant taxa [4, 14, 16, 38, 39, 51]. As the ICI results suggest, the ectoparasites *N. pacifica*, *Pr. manteri*, and *Ca. robustus* play an important role in structuring the parasite infracommunities of *C. sexfasciatus*. Due to ease of transmission through direct contact, ectoparasite populations contribute substantially to the structuring of infracommunities in gregarious fish such as carangids [23, 29, 31]. Fluctuations in the infection levels of some endoparasite species (e.g. *B. varicus* and the Tetraphyllidean cestode) can be attributed mainly to changes in *C. sexfasciatus* diet or foraging habitat, both between sampling years and climatic seasons (dry/rainy). Host body size was an important predictor of the total parasite load, as suggest by the global positive correlation (*r* = 0.633) recorded for this variable in the PCA (Table 3). In marine fish, body size has proved to be the main predictor of total parasite abundance [23, 39, 40]. A large body size can facilitate parasite colonization, since larger individuals tend to ingest greater quantities of food and are older and thus have had more time to accumulate parasites than smaller individuals [39, 40]. However, the negative correlations registered between host body size and species richness and diversity parameters suggest that the richest and most diverse parasite infracommunities were recorded in smaller rather than larger fish. Ontogenic changes in the feeding habits [10, 11, 20, 51] of *C. sexfasciatus* can explain the broad dietary diversity in smaller fish (for example, October 2016 and November 2018 samples, Table 2). This would in turn raise infracommunity species richness and diversity in these individuals (Table 2), as indicated in the diet analysis. Parasites are known to be useful as biological tags in distinguishing between fish stocks of the same species [47, 54], but the parasite fauna method has rarely been used to quantify possible variations in community structure and species composition over time. The discriminant analysis results (Table 4) indicated that the high variation in the infection levels of at least five parasite species (the monogeneans *N. pacifica* and *Pr. manteri*; the digenean *E. virgulus*; and the copepods *Ca. alalongae* and *L. ilishae*) may generate substantial changes in the parasite community structure of *C. sexfasciatus* over time. In other words, even though community species composition remains relatively stable, its structure may be less predictable.

Overall, the parasite communities of *C. sexfasciatus* were characterized by high numerical dominance of ectoparasites, mainly monogenean species. Community structure and species composition varied between sampling years and climatic seasons. Despite occurrence of a distinctive set of host-specialist parasites (monogenean species), similarity between the component parasite communities was generally low. Seasonal or local variations in some biotic (e.g. feeding behavior, body size, and infected prey availability) and abiotic environmental factors are possible sources of the observed interannual variations in *C. sexfasciatus* parasite community structure and species composition.
